# Assessment of service quality of public antiretroviral treatment (ART) clinics in South Africa: a cross-sectional study

**DOI:** 10.1186/1472-6963-12-228

**Published:** 2012-07-31

**Authors:** Hans F Kinkel, Adeboye M Adelekan, Tessa S Marcus, Gustaaf Wolvaardt

**Affiliations:** 1 Department of Family Medicine, University of Pretoria, P/Bag x323, Arcadia, Pretoria 0007, South Africa; 2 Foundation for Professional Development, P.O. Box 75324, Lynnwood Ridge, Pretoria 0040, South Africa; 3 Centers for Disease Control and Prevention, P.O. Box 26256, Arcadia, Pretoria, 0007, South Africa

## Abstract

**Background:**

In South Africa the ever increasing demand for antiretroviral treatment (ART) runs the risk of leading to sub-optimal care in public sector ART clinics that are overburdened and under resourced. This study assessed the quality of ART services to identify service areas that require improvement.

**Methods:**

A cross-sectional study was carried out at 16 of 17 public ART clinics in the target area in greater Pretoria, South Africa. Trained participant observers presented as ART qualifying HIV positive patients that required a visit to assess *treatment readiness*. They evaluated each facility on five different occasions between June and November 2009, assessing the time it took to get an appointment, the services available and accessed, service quality and the duration of the visit. Services (reception area, clinician’s consultation, HIV counselling, pharmacy, nutrition counselling and social worker’s assessment) were assessed against performance standards that apply to all clinics. Service quality was expressed as scores for clinic performance (CPS) and service performance (SPS), defined as the percentage of performance standards met per clinic and service area.

**Results:**

In most of the clinics (62.5%) participant observers were able to obtain an appointment within one week, although on the day of their visit essential services could not always be accessed. The median CPS of the assessed facilities was 68.5 with four clinics not meeting minimum standards (CPS > 60). The service areas that performed least well were the clinician’s consultation (SPS 67.3) and HIV counselling (SPS 70.7). Most notably, clinicians performed a physical examination in only 41.1% of the visits and rarely did a complete TB symptom screening. Counsellors frequently failed to address prevention of HIV transmission.

**Conclusions:**

Overall public sector ART clinics in greater Pretoria were easily accessible and their services were of an acceptable quality. However, the time spent at the clinic to complete the services was found to be very long and there was considerable variation in adherence to performance standards within the services, particularly in respect of clinician’s consultation and counselling. Clinic management needs to ensure efficient clinic organisation and to improve adherence to performance standards in key service areas.

## Background

South Africa has one of the highest burdens of HIV infections in the world with an estimated prevalence in 2009 of 17.8% in the adult population [1]. Since the start of antiretroviral treatment (ART) roll out in the public sector in 2003 almost one million people were estimated to be on ART by the end of 2009 [2,3]. This number represents approximately 37% of the population eligible for ART initiation based on WHO guidelines [3].

While several studies have shown ART to be effective in reducing HIV related deaths in South Africa [4-10], there is only limited research on the quality of ART service delivery in the country. Research in clinics in the Free State found high patient satisfaction with ART services, notwithstanding discontent with human resource shortages, overburdened staff and long waiting times [5]. In Gauteng, a comprehensive evaluation of two community health centres (CHC) and two hospital-based chronic care management and treatment (CCMT) sites providing ART found that high quality chronic care was being delivered in the public health system [11], although there were problems with both space and staff shortages. This resulted in long waiting lists, some patients being turned away without drugs, inadequate follow-up testing and failure to trace patients lost to follow-up. The study identified staff burnout and dissatisfaction as the major threat to quality of care. More recently, a study in Cape Town found that the service package for pre-ART care was not being fully implemented, resulting in gaps in the quality of care and missed opportunities for integrated care and positive prevention [12].

Guidelines and performance standards to define quality ART service provision have been developed at national and international level [13,14]. They set out what is expected from health care workers at each of the respective service areas (reception, front station, clinician’s consultation, HIV counselling, nutritional counselling, social worker’s assessment and pharmacy) on the various occasions that patients use their services. It has been argued that patient volume, limited funding, the shortage of health care workers, a generally overburdened public health care system and other factors make these standards difficult to meet in resource-limited settings like South Africa [5,15-20].

The objective of this study was to use performance standards to evaluate the quality of ART services provided at ART clinics in and around Pretoria in order to define service areas that required improvement and to assist clinic management to target interventions for quality improvement.

## Methods

Seventeen ART clinics in Tshwane district, Gauteng Province, South Africa and neighbouring districts participated in the study between June and November 2009. The ART clinics were selected based on their geographical location in the greater Pretoria area and that they could be accessed using public transport. Eleven patients were recruited from a NGO run clinic as participant observers. They carried out the assessment of ART services at the remaining 16 government clinic sites. To ensure confidentiality and anonymity, the clinics were randomly given a number between 1 and 16.

The clinics in the study did not differ from one another in terms of the way they organized and executed the services they provided. They all used the National Department of Health’s *Performance Standards for Antiretroviral Therapy*[13] a tool that outlines the procedural requirements for the *ART initiation* and subsequent *Follow-up* visits.

According to this guideline, a patient who qualifies for ART (based on the patient’s immune status or clinical condition) first needs to be assessed for *treatment readiness* ( Additional file 1 Textbox 1). The second visit is the *ART initiation* visit when the patient is supposed to start ART treatment.

All staff members were trained on and were qualified to meet these performance standards prior to being appointed at their respective.

As an investigation of services offered on the *treatment readiness* visit, the study assessed the quality of services provided at the reception area and front station as well as during the clinician’s consultation, HIV counselling, nutrition counselling, social worker’s assessment and pharmacy (optional). Data were collected using a checklist based on performance standards for each service area. Table1 sets out the performance standards that were assessed. In addition, data were collected on how quickly an appointment at the facility could be made by the participant observers on telephonic request, the time spent at the facility overall and the time each participant observer spent at each of the service areas during his or her appointment. Basic frequency analysis was done using Microsoft® EXCEL.

**Table 1:** Percent of performance standards (PS) fulfilled across all assessed facilities

**Service area**	**n**	**Percent PS fulfilled**
**Reception (admin clerk)**		
Opens a file for the patient	69	98.6
Checks if patient has a referral letter	70	95.7
Checks if patient brought lab result	70	91.4
Shows patient where to go next	69	85.5
Greets patient	70	74.3
Immediately shows patient to the nurse	68	69.1
Confirms booking	69	59.4
**Front station (nurse)**		
Checks blood pressure	66	87.9
Measures weight	68	80.9
Checks pulse	67	79.1
Tells patient where to go next	67	76.1
Greets patient	68	66.2
Checks temperature	65	63.1
Measures height	66	39.4
**Clinician’s Consultation (medical doctor)**		
Reviews lab results	54	87.0
Asks about previous TB history	59	86.4
Tells patient where to go next	57	82.8
Asks about alcohol, smoking and other drugs	57	80.7
Greets patient	59	79.7
Determines timeframe for follow-up visit	58	79.3
Asks about previous diseases	57	78.9
Asks about previous medication	58	75.9
Asks about loss of weight	58	72.4
Asks about concomitant medications (e.g. herbal medication)	58	72.4
Asks about the use of any prophylaxis	57	71.9
Asks about coughing	57	66.7
Verifies previous exposure to antiretroviral drugs	58	60.3
Asks about night sweat	59	57.6
Asks about difficulty in breathing	59	52.5
Checks psycho-social condition	54	51.9
Confirms/excludes pregnancy (women only)	43	46.5
Performs physical examination	56	41.1
Requests to bring all concurrent medications at the next visit	56	37.5
Refers for cervix cancer screening smear (women only)	34	35.3
**HIV counselling (lay counsellor)**		
Greets patient	49	87.8
Reinforces the importance of using ART always at the same time	49	83.7
Provides the information that ART may cause adverse effects	48	83.3
Tells patient where to go next	46	78.3
Provides information that ART is a combination of medicine	49	77.6
Provides the information that ART doesn't cure AIDS but prolong life	49	77.6
Completes form	47	74.5
Discusses the importance of avoidance of alcohol and other drugs	49	73.5
Discusses prevention of HIV super-infection	46	69.6
Explains the importance of adherence with regard to resistance	49	69.4
Assesses patient’s knowledge of ART	48	68.8
Discusses the importance of proper nutrition	48	66.7
Discusses ART and how antiretroviral drugs work	49	65.3
Advises patient not to stop medicines without talking to clinician	49	65.3
Advises not to start any new medicines without consultation	49	65.3
Discusses prevention of HIV infection of others	48	64.6
Recommends the use of reminder tools for use of ART	48	62.5
Discusses the importance of physical activities	49	61.2
Shows types of ART as samples	49	53.1
Discusses PMTCT (women only)	34	50.0
Advises patient not to share medication with others	49	46.9
**Social worker’s assessment (social worker)**		
Confirms contact information	9	100.0
Greets patient	9	88.9
Tells patient where to go next	9	88.9
Assesses alcohol and other drug use	10	80.0
Assesses housing	10	80.0
Assesses access to transportation	10	70.0
Verifies if patient qualify for grant application	10	70.0
Assesses need for food supplements	10	70.0
Assesses social violence	9	44.4
**Nutrition counselling (dietician)**		
Greets patient	10	90.0
Performs nutritional evaluation	10	90.0
Tells patient where to go next	10	90.0
Provides dietary education	10	90.0
Completes nutritional risk score	10	80.0
Provides basic knowledge of food security	10	70.0
**Pharmacy (pharmacist/pharmacy assistant)**		
Reviews the prescribed drugs	37	91.9
Counsels patient on treatment	37	83.8

n: number of assessments by participant observers; PS: performance standards.

The criteria used for selecting participant observers were that they had previous exposure to a public ART clinic, had sufficient understanding of the intention of the evaluation and had similar socio-demographic characteristics as the patient population served by public ART clinics. Also they had to be mobile and available to make multiple clinic visits. Participant observer training lasted approximately three hours and was conducted by the research team in one on one or small group sessions. It included a briefing on the purpose of the study, the provision of fictitious blood results that would qualify a participant observer to start ART, an explanation of the checklist used to screen adherence to performance standards and an instruction on how to document the findings. Participant observers were instructed to act as patients and not to disclose their research role. They also were encouraged to fill out the checklist as soon as possible after completing a service in order to minimize recall bias. After conducting each evaluation, participant observers were interviewed to determine the validity of their experiences. They were asked about their experience at the clinic and the answers in the checklist were reviewed. To ensure that the checklist worked and that the participant observers recorded meaningful information the method was tested twice prior to carrying out the assessment.

Each study clinic agreed to be assessed by participant observers on their adherence to the ART performance standards with the understanding that they would not be made aware of the timing of the evaluation nor would they be told who the actual participant observers were. The plan was to generate 80 assessments, with each clinic being assessed on five different days by different participant observers. Due to the limited availability of some participant observers, in practice it was only possible to complete a total of 70 evaluations.

Data about the total number of patients that visited the clinic in the months of assessment and the workload per staff was collected from clinic registers. Workload per staff was calculated as the total number of patients that visited the clinic in the month of the assessment divided by the full time equivalent (FTE) per staff in that month. It was not possible to disaggregate the volume of patients by the reason for or type of visit as this information was not reliably collected at the assessed clinics.

The results were analyzed for each facility in the following way. The services that were available at the clinics and which services participant observers were able to access were determined. Then an overall clinic performance score (CPS) of between 0 and 100 was calculated for each facility. The CPS was defined as the percentage of performance standards met by all services provided in the facility. To estimate the overall quality of service provided in the 16 assessed facilities the median of the CPS was calculated. Similarly a service performance score (SPS) was calculated for each service area at each facility, defined as the percentage of performance standards met by a particular service area per facility as assessed by the participant observers. To estimate the overall quality of a service area across all clinics, the median of the SPS was calculated.

In order to define service performance gaps across all the clinics, each performance standard was looked at individually and the percentage value of the particular performance standard being met across all sites was calculated. According to the percent of performance standards met, performance was ranked as excellent (≥ 90%), very good (80% - 89%), good (70% - 79%), acceptable (60% - 69%) and unacceptable (< 60%).

The overall time spent at each facility was calculated as the median of the times spent per visit at the clinic.

The possibility that the high workload of clinicians or counsellors affected their adherence to performance standards, *inter alia*, reduced the time they spent with patients and affected the quality of the services they provided, was also analysed. We therefore sought to determine if the workload of clinicians and counsellors during the month of the visit was a predictive factor for the duration of the visit, and if the duration of the visit was a predictive factor for the quality of the service. This was derived from a linear regression analysis (Microsoft® Excel, version 2007) of the information available for each visit where the correlation coefficient *R*^*2*^ was used to describe the association. Accordingly, the closer *R*^*2*^ values were to zero the lesser the association, the closer the values were to one the higher the correlation.

This research was approved by the Faculty of Health Sciences Research Ethics Committee of the University of Pretoria, South Africa (Protocol number 75/2011) and conducted in compliance with the Helsinki Declaration.

## Results

### Clinics

Of the 16 clinics assessed, two were situated in the city centre of Pretoria, six were in former townships and eight clinics were in rural areas. Table2 shows the total number of patients per facility per months in which the assessments took place and the workload per clinician and HIV counsellor.

**Table 2:** Total number of patients per month per facility and workload per staff (clinician and counsellor)

**Facility**	**Month of assessment**	**Total number of patients**	**No. clinicians**	**No. patients per clinician**	**No. counsellors**	**No. patients per counsellor**
1	Aug	2783	6	464	13	214
	Oct	3118	6	520	11	283
2	Aug	616	1	616	3	205
	Oct	987	1	987	2	494
3	Jul	1087	1	1087	3	362
	Oct	1343	1	1343	3	448
4	Jul	1550	1	1550	4	388
	Oct	2110	1	2110	4	528
5	Sep	855	2	428	3	285
	Oct	876	2	438	3	292
6	Jul	3661	10	366	9	407
7	Jun	n.d.	n.d.	n.d.	n.d	n.d.
	Oct	2765	5	553	17	163
8	Aug	2008	3	669	5	401
	Oct	2145	3	715	5	429
9	Aug	2809	5	562	11	255
	Oct	3121	5	624	11	284
10	Sep	1507	1.6	942	3	502
	Oct	1331	1	1331	3	444
	Nov	1571	1	1571	3	524
11	Jun	2886	5	577	4	722
	Oct	3460	5	692	4	865
	Nov	3479	5	696	4	870
12	Jun	1422	2	711	4	356
	Aug	1426	2	713	4	357
	Nov	1903	2	952	4	476
13	Oct	1087	1	1087	3	362
	Nov	1357	1	1357	3	452
14	Sep	1009	2	505	5	202
	Oct	948	1	949	5	190
	Nov	933	2	467	5	187
15	Sep	682	2	342	4	171
	Oct	833	2	417	4	208
	Nov	798	2	399	4	200
16	Aug	2065	2	1033	8	258
	Nov	1773	2	887	8	222
**Median**		**1507**		**696**		**357**
**IQR**		**1457**		**498**		**232**

No.: number, n.d.: no data available, IQR: interquartile range, Source: clinic registers.

### Bookings

**Figure 1:**
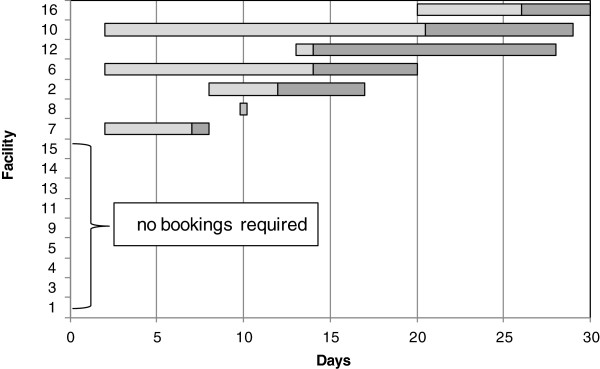
**Median duration until appointment per facility in days.** The left end of the light grey bar indicates the minimum, the right end of the light grey bar indicates the median and the right end of the dark grey bar the maximum of days until appointment at a particular facility.

In nine clinics (56.3%) no booking was required and the patients could access the facility on any day. The remaining seven clinics required bookings, with a median of 14 days until appointment (interquartile range [IQR]: 11.5 days) (Figure1).

### Services offered

**Figure 2:**
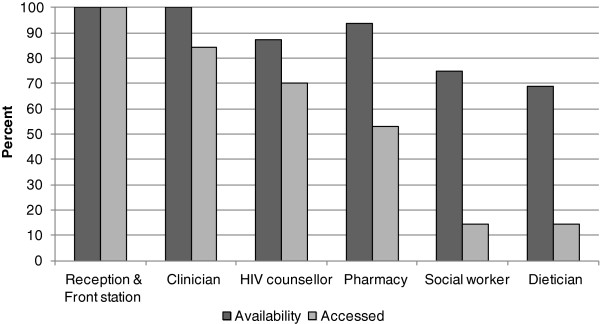
Service availability at the facilities and services accessed by participant observers.

Almost all clinics provided core services (reception and front station, clinician’s consultation, HIV counselling and pharmacy). Adjunct services, such as social worker assessment and nutrition counselling, were offered in 75% and 68.8% of the facilities, respectively (Figure2).

### Services accessed

Access to services was influenced by their general availability at the clinic and their actual availability on the day of visit. On the 70 clinic visits, participant observers were able to access the reception and front station on all occasions (100%), the clinician on 59 (84.3%) visits, the HIV counsellor on 49 (70%) visits, the pharmacy on 37 visits (52.9%), the social worker on 10 visits (14.3%) and the dietician on 10 visits (14.3%) (Figure2).

### Quality of service

The median of the clinic performance scores (CPS) for the 16 assessed facilities was 68.5 (IQR: 18.7). However, one quarter of the clinics did not meet minimum standards (CPS < 60). Across all the facilities, the services with the lowest performance scores were clinician’s consultation (SPS 67.3) and HIV counselling (SPS 70.7) (Table2).

### Quality gaps

Quality gaps refer to the extent to which standards internal to the service were met during a visit. A detailed analysis was made of the quality gaps in the clinician’s consultation and HIV counselling, the two least performing services that are also the two most essential services. It revealed that clinicians did a physical examination in only 41.1% of the visits. As part of their screening for TB they asked questions about difficulties in breathing and the presence of night sweats or a cough in 52.5%, 57.6% and 66.7% of the consultations, respectively. Counsellors addressed PMTCT with female patients in only 50% of consultations and the prevention of HIV infection to others in only 64.6% (Table3).

**Table 3:** Service performance scores (SPS) and clinic performance score (CPS) per facility

**Facility**	**Service performance score (SPS)**												**Clinic performance score (CPS)**
	**Reception & Front station**		**Clinician’s consultation**		**HIV counselling**		**Pharmacy**		**Social worker’s assessment**		**Nutrition counselling**		
	**SPS**	**n**	**SPS**	**n**	**SPS**	**n**	**SPS**	**n**	**SPS**	**n**	**SPS**	**n**	
1	71.4	5	87.5	4	65.7	5	75.0	4	n.d.		66.7	1	74.0
2	100	5	92.0	5	86.7	5	100	3	85.2	3	94.4	3	91.7
3	69.5	5	63.2	1	n.d.		n.d.		n.d.		n.d.		67.9
4	60.3	5	34.2	4	90.9	1	100	3	n.d.		n.d.		54.3
5	94.0	5	88.4	5	77.8	5	100	4	93.8	2	100	1	86.9
6	83.9	4	70.7	4	n.d.		100	1	n.d.		n.d.		76.7
7	81.8	5	65.8	5	32.5	3	0.0	1	n.d.		n.d.		62.4
8	95.2	3	96.5	3	54.8	2	100	2	n.d.		n.d.		84.1
9	72.7	4	50.7	4	58.3	3	75.0	2	28.6	1	n.d.		58.8
10	62.3	5	34.5	3	76.5	4	100	1	n.d.		n.d.		60.5
11	60.7	3	68.9	3	70.0	3	66.7	3	11.1	1	0.0	1	63.2
12	69.6	4	60.7	3	71.4	3	83.3	3	n.d.		100	1	69.0
13	50.0	3	40.0	3	25.0	2	75.0	2	n.d.		n.d.		39.7
14	61.8	4	33.3	3	67.5	4	100	1	n.d.		100	1	57.6
15	92.5	5	74.3	4	75.9	4	100	2	100	1	100	1	82.0
16	85.7	5	71.1	5	72.5	5	100	5	88.9	2	100	1	77.7
**Median**	**72.1**		**67.3**		**70.7**		**100.0**		**87.0**		**100.0**		**68.5**
**IQR**	**25.2**		**29.6**		**16.2**		**25.0**		**49.8**		**12.5**		**18.7**

n: number of assessments by participant observers, n.d.: no assessments done, IQR: interquartile range.

### Time

**Figure 3:**
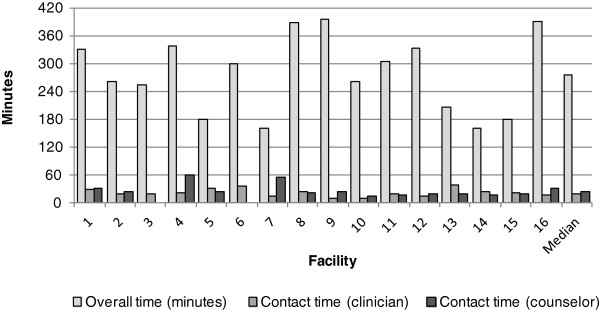
Median total time participant observers spent at the facility and contact time participant observers spent with the clinician and HIV counsellor.

Overall, the median time participant observers spent at the clinics for a first visit appointment was 4.6 hours (IQR: 3.15 hours). The median time they spent with the clinician was 20 minutes (IQR: 15 minutes) and with the counsellor, 25 minutes (IQR: 28 minutes) (Figure3).

### Factors predicting service performance

**Figure 4:**
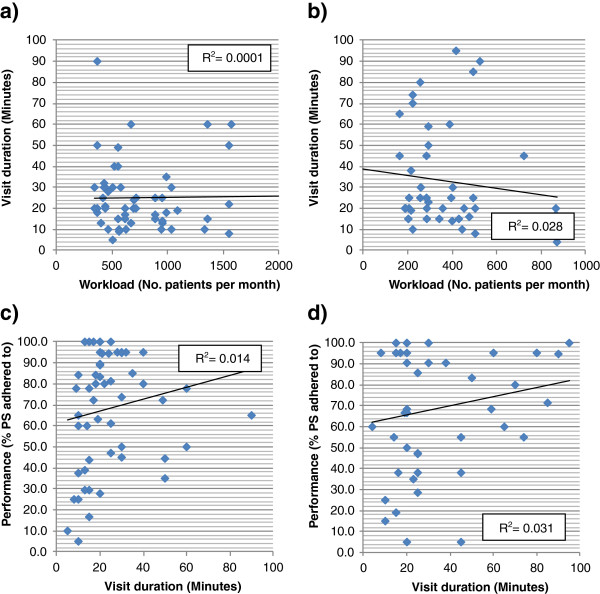
**Correlation of the workload and the duration of the visits per staff (Figure 4a [clinicians] and 4b [counsellors]), and correlation of the duration of the visits and performance per staff (Figure 4c [clinicians] and 4d [counsellors]).** Each dot (♦) represents a particular visit; R^2^: correlation coefficient; PS: performance standards.

A comparison of the workload of the clinician or counsellor with the time they spent in contact with participant observers revealed that a lower number of patients seen per month per staff did not necessarily translate into longer consultation times (Figure4a and 4b). Further, a correlation of consultation contact times with the quality of the clinician or counsellor care revealed that longer consultation times did not necessarily mean better performance or care (Figure4cB and 4).

## Discussion

This cross-sectional study assessed access to and quality of services in 16 ART clinics in greater Pretoria between June and November 2009. The CPS, as the overall measure for service quality at the facilities, was 68.5%. This means that, on average, about two thirds of the expected performance standards were fulfilled at the clinics assessed. While this average is suggestive of an acceptable general result, it conceals the fact that the CPS varied markedly among the facilities ranging from 39.7% to 91.7%. Four clinics did not meet minimum standards (60% of the expected performance standards), while only one clinic performed excellently (CPS > 90).

Deeper analysis of the results on service quality paints an even less satisfactory picture. A comparison of the various service areas across the clinics reveals that the most essential services, the clinician’s consultation and HIV counselling had the weakest performance. With a median SPS of 67.3 and 70.7, respectively, they scored markedly lower than social worker assessment, pharmacy and nutrition counselling. Especially, clinician consultations varied markedly in their adherence to performance standards at the assessed clinics. In five clinics, clinicians failed to adhere to half of the performance standards, while in four others clinicians adhered to more than 80% of the expected performance standards. One reason for the overall poor performance of clinicians and HIV counsellors, when compared to the other services at the clinic, is the higher number and more sophisticated nature of the expected performance standards of clinicians and counsellors. Arguably, these could make their services more sensitive to pressures of time and patient volume at the clinics. The findings here suggest, however, that the problem is deeper. An examination of each service area’s expected performance revealed the clinician consultation to have a high number of shortfalls in core performance areas. The most worrisome of these were the low rates of physical examination (41.1%) and failure to do comprehensive TB screening. Although clinicians almost always asked about previous TB treatment, they underperformed in assessing actual TB signs and symptoms. Particularly, assessment of difficulty in breathing and questions about the presence of night sweats, cough and weight loss was done in only half of the consultations. Also, while most clinicians took a good drug history, which included questions about previous medication, the concomitant use of herbal/traditional medication and the use of any prophylaxis, they often failed to ask about previous exposure to antiretroviral drugs. Further, a psychosocial history was taken in only half of the consultations. Not even half of the female patients were asked if they were pregnant, and even fewer were referred for cervical cancer screening. Thus, many clinicians failed to get all the information they would need to decide upon the further clinical management of their patients and eventually to determine the choice of the ART regimen. The observations also reveal that apparently important opportunities for integration of care (TB and cervix cancer screening) were missed.

As with clinician performance standards there were also many shortcomings in HIV counselling. Counsellors infrequently assessed patient knowledge about HIV. They mostly did not provide basic information about ART, such as showing pill samples, explaining how antiretroviral drugs work and discussing the importance of treatment adherence. Counsellors also did not consistently address the practical issues of taking ART, such as advising patients not to share medication with other patients and to not start or stop ART without consulting a clinician. Similarly, preventive measures, such as PMTCT and how to prevent infection to others as well as advice on the importance of physical activity, proper nutrition and other life style issues were infrequently addressed. These findings suggest that many patients may not be well prepared for ART, especially key aspects of treatment adherence. Also, the opportunity to address HIV transmission was frequently missed.

By contrast, the front station satisfactorily met its performance standards, except that nurses rarely measured height, inconsistently took temperatures and often didn’t greet patients. Although greeting a patient may appear to be of less importance as a performance standard compared to clinical standards of care, it may impact on the acceptance of the services provided by patients and the community.

Almost all performance standards for the reception, the pharmacy and nutritional counselling were met at a good rate (> 70%), as were the performance standards for the social worker consultations, except with regard to interpersonal, gender based violence, which was addressed in only half of the consultations.

These results point in the same direction as the findings of a recently published retrospective study in Cape Town, which assessed the quality of care during the pre-ART period (the time between HIV counselling and testing until initiation of ART). Although different in approach, the study found similarly low rates of screening for TB symptoms or cervical cancer as well as missed opportunities to integrate care and prevention [12].

Time spent in getting the services is also an important indicator of quality of service at ART clinics as it impacts on the acceptance of the services. Time here is understood as both overall time spent at the clinic and time spent in actual service contact. In this study, the median overall time of 4.6 hours spent by participant observers at a *treatment readiness* assessment visit was substantially longer than the NDOH’s target of 3.8 hours [21]. However, the median contact time of 20 minutes with the clinician and 25 minutes with the HIV counsellor, the two key services of an ART clinic, were not even half the NDOH targeted time of 45 and 60 minutes, respectively. This finding goes a long way to explaining the less than satisfactory performance of clinician and counsellor standards, as no matter their intentions, they did not have enough actual time to complete the tasks expected of them.

The longer overall time spent at clinics and the shorter time spent with the clinician or counsellor suggests an overburdened system. This finding is supported by the fact that in almost all clinics the actual number of patients per month per clinician and counsellor exceeds the NDOH benchmark of 500 patients per month per clinician and 100 patients per month per counsellor [21].

At the same time, a lower number of patients seen per month per staff at a clinic did not necessarily translate into longer consultation times. In other words, some facilities managed clinician and counsellor contact times in a way that allowed them adequate time to consult, despite very high patient loads, while others with lower patient loads did not. This result highlights the importance of organization and management in service quality.

This said, the finding that longer consultation times did not necessarily result in better performance also points to another critical factor in determining performance standards, namely, that of individual professional performance. Less than optimum quality of care in the clinician’s consultation and HIV counselling was not just a matter of time constraints. It was also determined by the individual health care worker’s skills and performance. Given available training, this problem is likely to be less a matter of knowledge and more a matter of attitude and motivation. Limited available research in Gauteng suggests that burn out and dissatisfaction have saturated staff capacities at clinics [11].

The quality of ART services in South Africa is not only determined by staff performance at the clinics, as measured by the adherence to performance standards and the time spent in getting the services. It is also gauged by the general accessibility and availability of such clinics. While waiting times of up to several months to get an appointment at an ART clinic were common in South Africa only a few years ago, this problem has been unequivocally overcome in greater Pretoria and probably in many other urban areas as well [17,22,23]. This study found ART services to be generally easily accessible in the study area. It was greatest at nine clinics where patients could walk in and be attended to without prior booking while most patients got an appointment within 14 days in the seven clinics where bookings were required. This finding is in line with the countrywide trend towards greater access to ART services.

At the same time, this study suggests that the ability of patients to access the full spectrum of ART services to assess *treatment readiness* was also a factor of clinic functioning. Services considered essential to the determination of ART initiation, especially consultation by a clinician and HIV counselling, were not always accessed. Those that might be considered more optional, at least on the first visit, such as pharmacy services, social worker assessments and nutritional counselling were even less available (52.9%, 14.3% and 14.3%). The reasons for the non-availability of especially core, but also adjunct services would require further investigation. This notwithstanding, incomplete and/or multiple ‘initial’ assessment visits is likely to have direct negative consequences for patients, including delayed ART initiation. It also carries negative implications for the clinics, potentially contributing to patient dissatisfaction with their services.

The design of this study, using participant observers as assessors, allowed for an evaluation of the facilities in their day-to-day operations. However, some limitations have to be considered when interpreting the results. The relatively small number of observations (n = 70) limits the ability to generalise the findings beyond the sites investigated. Also, small clinics with low patient volumes were over-represented in analyses across all clinics. And there is the possibility that the assessments of the participant observers were influenced by recall and interpretation bias. This said, we believe that these results, based on participant observer assessments against defined performance standards, provided a deeper insight into the reality of patient care in public ART clinics than patient satisfaction surveys that assess the service quality.

## Conclusions

In conclusion, this study of first appointment (*treatment readiness*) performance standards of ART clinics in and around Pretoria presented a mixed picture of the quality of services. ART clinics were easily accessed and were of an overall acceptable quality. However, they showed long waiting times and variability within services, especially in the key areas of clinician’s consultation and HIV counselling. These findings suggest that there is a need to improve service performance. In this regard, particular attention should be paid to the time patients spend at facilities, and especially the time they spend in clinical consultations and counselling. Patients would benefit from a review by facility management of their approach to several areas of clinic functioning, especially general access (for example, by having a booking system, and by differentiating between first and follow-up appointments), operational flow (in order to ensure adequate time for patient contact with key service stations) and staff training, practice and development. The vexing problem of ensuring quality ART services is likely to continue to dog a system where ART services are widely available and easily accessible (at least in most urban [23], although not in most rural [22] areas of South Africa) but capacities are saturated. Lastly, participant observation measured against performance standards provided novel insights into the issues of quality of ART care in a primary care setting that previously have not been obtained.

## Competing interests

The authors declare that they have no competing interests.

## Authors' contributions

HFK conceptualized and designed this study, performed the statistical analyses, interpreted the data and drafted the manuscript. AMA participated in the design of the study, coordinated the participant observers (recruitment, training, sending out and debriefing), collected and captured the data and participated in the performance of statistical analyses and interpretation of the data. TSM has helped to draft the manuscript and revised the manuscript critically for important intellectual content. GW conceptualized the study, participated in the design and has revised the manuscript critically for important intellectual content. All authors read and commented on drafts of the article and approved the final version.

## Pre-publication history

The pre-publication history for this paper can be accessed here:

http://www.biomedcentral.com/1472-6963/12/228/prepub

## Supplementary Material

Additional file 1 **Textbox 1.***Treatment readiness* visit. Click here for file
