# Supplementary material for: Assessment of service quality of public antiretroviral treatment (ART) clinics in South Africa: a cross-sectional study
Source: BMC Health Serv Res. 2012 Jul 31;12:228. doi: 10.1186/1472-6963-12-228 (PMC3468362; doi:10.1186/1472-6963-12-228)
Supplement: Additional file 1 — Textbox 1.Treatment readiness visit. [file 1472-6963-12-228-S1.pdf]

The initial *treatment readiness* visit involves the following service areas and procedures
• Reception: an administrative clerk registers the patient and opens a file<br>• Front station: a nurse triages the patient and measures vitals<br>• Clinician's consultation: a medical doctor takes the medical history of the patient, examines the patient and develops the diagnostics and treatment plan<br>• Laboratory: a phlebotomist draws blood for routine baseline tests<br>• HIV counselling: a trained lay counsellor informs the patient about general aspects of ART and provides advice on ART drugs, treatment adherence and HIV prevention strategies<br>• Social worker's assessment: a social worker assesses the patient's social situation<br>• Nutrition counselling: a dietician informs and advises the patient about healthy nutrition<br>• Pharmacy (optional): if a patient is prescribed medication, a pharmacist or a supervised pharmacy assistant dispenses the drugs and provides information on how to take them
